# 1,3-Bis{[(4-methyl­phen­yl)sulfon­yl]­oxy}propan-2-yl 4-methyl­benzene­sulfonate

**DOI:** 10.1107/S1600536812008227

**Published:** 2012-02-29

**Authors:** Yusrabbil Amiyati Yusof, Azhar Ariffin, Hazimah Abu Hassan, Seik Weng Ng, Edward R. T. Tiekink

**Affiliations:** a Department of Chemistry, University of Malaya, 50603 Kuala Lumpur, Malaysia; b Malaysian Palm Oil Board, Bandar Baru Bangi, 43000 Bangi, Malaysia; c Chemistry Department, Faculty of Science, King Abdulaziz University, PO Box 80203 Jeddah, Saudi Arabia

## Abstract

In the title sulfonate derivative, C_24_H_26_O_9_S_3_, all atoms apart from those of one of the 4-methyl­benzene­sulfonate residues lie approximately in a disc; the dihedral angles between the approximately orthogonal benzene ring and those in the plane are 74.53 (9) and 67.79 (11)°. In the crystal, mol­ecules are consolidated into the three-dimensional architecture by C—H⋯O inter­actions. One of the 4-methyl­benzene­sulfonate residues is disordered over two almost parallel positions; the major component refined to a site-occupancy factor of 0.918 (2).

## Related literature

For use of the title compound as a stabilizer for thermal recording materials, see: Matsumoto *et al.* (1996 ▶). For a related structure, see: Al-Mohammed *et al.* (2011 ▶).
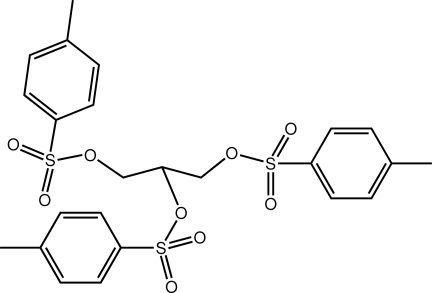


## Experimental

### 

#### Crystal data

C_24_H_26_O_9_S_3_
*M* *_r_* = 554.63
Triclinic, 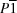

*a* = 7.6887 (3) Å
*b* = 12.9635 (5) Å
*c* = 13.6887 (5) Å
α = 98.943 (3)°
β = 100.292 (3)°
γ = 105.174 (3)°
*V* = 1265.91 (8) Å^3^
*Z* = 2
Cu *K*α radiation
μ = 3.13 mm^−1^
*T* = 100 K
0.35 × 0.30 × 0.25 mm

#### Data collection

Agilent SuperNova Dual diffractometer with an Atlas detector
Absorption correction: multi-scan (*CrysAlis PRO*; Agilent, 2011 ▶) *T*_min_ = 0.407, *T*_max_ = 0.508
9241 measured reflections
5191 independent reflections
4801 reflections with *I* > 2σ(*I*)
*R*_int_ = 0.018

#### Refinement

*R*[*F* ^2^ > 2σ(*F* ^2^)] = 0.035
*wR*(*F* ^2^) = 0.100
*S* = 1.06
5191 reflections
363 parameters
22 restraints
H-atom parameters constrained
Δρ_max_ = 0.44 e Å^−3^
Δρ_min_ = −0.53 e Å^−3^

### 

Data collection: *CrysAlis PRO* (Agilent, 2011 ▶); cell refinement: *CrysAlis PRO*; data reduction: *CrysAlis PRO*; program(s) used to solve structure: *SHELXS97* (Sheldrick, 2008 ▶); program(s) used to refine structure: *SHELXL97* (Sheldrick, 2008 ▶); molecular graphics: *ORTEP-3* (Farrugia, 1997 ▶) and *DIAMOND* (Brandenburg, 2006 ▶); software used to prepare material for publication: *publCIF* (Westrip, 2010 ▶).

## Supplementary Material

Crystal structure: contains datablock(s) global, I. DOI: 10.1107/S1600536812008227/jj2124sup1.cif

Structure factors: contains datablock(s) I. DOI: 10.1107/S1600536812008227/jj2124Isup2.hkl

Supplementary material file. DOI: 10.1107/S1600536812008227/jj2124Isup3.cml

Additional supplementary materials:  crystallographic information; 3D view; checkCIF report

## Figures and Tables

**Table 1:** Hydrogen-bond geometry (Å, °)

*D*—H⋯*A*	*D*—H	H⋯*A*	*D*⋯*A*	*D*—H⋯*A*
C1—H1*A*⋯O2^i^	0.99	2.58	3.430 (2)	144
C3—H3*A*⋯O2^i^	0.99	2.39	3.230 (2)	143
C3—H3*B*⋯O9^ii^	0.99	2.53	3.377 (2)	144
C6—H6⋯O4^iii^	0.95	2.51	3.332 (2)	145
C9—H9⋯O5^iv^	0.95	2.50	3.166 (2)	127
C15—H15⋯O6^v^	0.95	2.53	3.409 (4)	154
C20—H20⋯O3^vi^	0.95	2.60	3.545 (2)	176
C24—H24*B*⋯O8^vii^	0.98	2.55	3.263 (3)	129

Symmetry codes: (i) 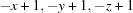; (ii) 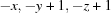; (iii) 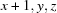; (iv) 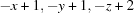; (v) 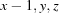; (vi) 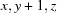; (vii) 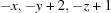.

**Fig. 1.**
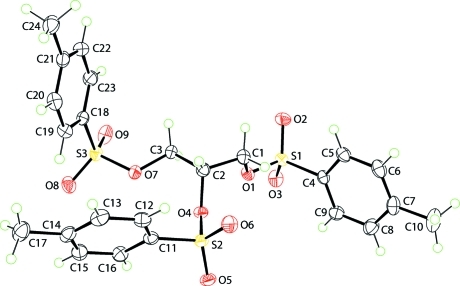
The molecular structure of (I) showing the atom-labelling scheme and displacement ellipsoids at the 50% probability level. Only the major component of the disordered residue is shown.

**Fig. 2.**
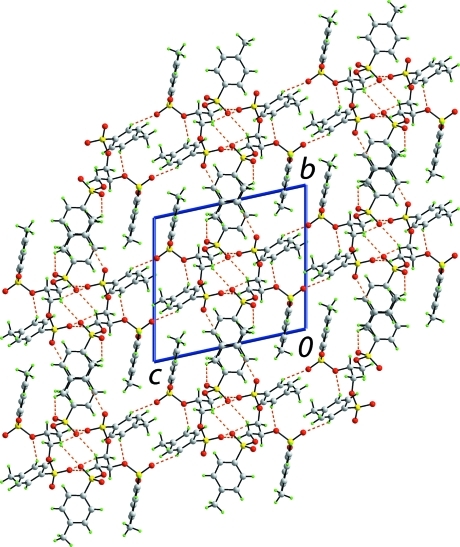
A view in projection down the a axis of the unit-cell contents of (I). The weak C—H···O interactions are shown as orange dashed lines.

## supplementary crystallographic information

### Comment

In continuation of the related structural studies (Al-Mohammed *et al.*, 2011), the crystal structure determination of the title compound, (I), is now described. Compound (I) has been patented as a stabilizer for thermal recording materials (Matsumoto *et al.*, 1996).

In (I), Fig. 1, each carbon of the propyl residue is connected to a 4-methylbenzenesulfonate residue. The S1- and S2-containing residues are approximately co-planar with the central propyl chain with the dihedral angle between their benzene rings being 51.00 (11)°. By contrast, the S3-containing 4-methylbenzenesulfonate projects almost orthogonally with respect to the remaining molecule. The dihedral angles between the benzene ring of the S3-residue and those of the S1- and S2- residues are 74.53 (9) and 67.79 (11)°, respectively.

Molecules are consolidated into the three-dimensional architecture by weak C—H···O interactions, Table 1. Globally, the crystal structure comprises alternating layers of sulfonate-rich and sulfonate-poor regions that stack along the *b* axis, Fig. 2.

### Experimental

Glycerol (5.53 g, 0.06 mol), pyridine (4 ml, excess) and *p*-toluenesulfonyl chloride (9.53 g, 0.05 mol) was stirred in dichloromethane (50 ml) and monitored by thin layer chromatography. On completion of the reaction, dilute hydrochloric acid was added and the product was purified by column chromatography. Crystals were obtained upon recrystallization from its *n*-hexane/ether solution.

### Refinement

Carbon-bound H-atoms were placed in calculated positions [C—H = 0.95 to 0.98 Å, *U*_iso_(H) = 1.2 to 1.5*U*_eq_(C)] and were included in the refinement in the riding model approximation.

The tosyl group connected to the middle carbon of the glycerol fragment is disordered over two positions, with the major component having a site occupancy factor = 0.918 (2). The 1,2- as well as the 1,3-related distances of the minor component were restrained to those of the corresponding distances in the major component. The pair of C_glycerol_–O_tolylsate_ distances were restrained to within 0.01 Å of each other. The anisotropic displacement parameters of the primed atoms were set to those of the unprimed ones.

### Crystal data

**Table:**

C_24_H_26_O_9_S_3_	*Z* = 2
*M**_r_* = 554.63	*F*(000) = 580
Triclinic, *P*1	*D*_x_ = 1.455 Mg m^−^^3^
Hall symbol: -P 1	Cu *K*α radiation, λ = 1.54184 Å
*a* = 7.6887 (3) Å	Cell parameters from 5203 reflections
*b* = 12.9635 (5) Å	θ = 3.4–76.0°
*c* = 13.6887 (5) Å	µ = 3.13 mm^−^^1^
α = 98.943 (3)°	*T* = 100 K
β = 100.292 (3)°	Prism, colorless
γ = 105.174 (3)°	0.35 × 0.30 × 0.25 mm
*V* = 1265.91 (8) Å^3^	

### Data collection

**Table:**

Agilent SuperNova Dual diffractometer with an Atlas detector	5191 independent reflections
Radiation source: SuperNova (Mo) X-ray Source	4801 reflections with *I* > 2σ(*I*)
Mirror monochromator	*R*_int_ = 0.018
Detector resolution: 10.4041 pixels mm^-1^	θ_max_ = 76.2°, θ_min_ = 3.4°
ω scan	*h* = −9→7
Absorption correction: multi-scan (*CrysAlis PRO*; Agilent, 2011)	*k* = −13→16
*T*_min_ = 0.407, *T*_max_ = 0.508	*l* = −16→17
9241 measured reflections	

### Refinement

**Table:**

Refinement on *F*^2^	Primary atom site location: structure-invariant direct methods
Least-squares matrix: full	Secondary atom site location: difference Fourier map
*R*[*F*^2^ > 2σ(*F*^2^)] = 0.035	Hydrogen site location: inferred from neighbouring sites
*wR*(*F*^2^) = 0.100	H-atom parameters constrained
*S* = 1.06	*w* = 1/[σ^2^(*F*_o_^2^) + (0.0582*P*)^2^ + 0.5065*P*] where *P* = (*F*_o_^2^ + 2*F*_c_^2^)/3
5191 reflections	(Δ/σ)_max_ = 0.001
363 parameters	Δρ_max_ = 0.44 e Å^−^^3^
22 restraints	Δρ_min_ = −0.53 e Å^−^^3^

### Fractional atomic coordinates and isotropic or equivalent isotropic displacement parameters (Å^2^)

**Table:**

	*x*	*y*	*z*	*U*_iso_*/*U*_eq_	Occ. (<1)
S1	0.49448 (5)	0.37561 (3)	0.65879 (3)	0.02049 (11)	
S2	0.51107 (6)	0.76065 (4)	0.89915 (3)	0.02057 (14)	0.9178 (19)
O4	0.40477 (17)	0.66335 (10)	0.80445 (9)	0.0204 (3)	0.9178 (19)
O5	0.46255 (18)	0.71611 (11)	0.98305 (10)	0.0256 (3)	0.9178 (19)
O6	0.70049 (18)	0.79885 (12)	0.89449 (10)	0.0289 (3)	0.9178 (19)
S2'	0.5610 (6)	0.8258 (4)	0.8805 (3)	0.02057 (14)	0.08
O4'	0.5398 (17)	0.7696 (8)	0.7667 (6)	0.0204 (3)	0.08
O5'	0.497 (2)	0.7436 (11)	0.9354 (11)	0.0256 (3)	0.08
O6'	0.7505 (13)	0.8922 (11)	0.9127 (10)	0.0289 (3)	0.08
C11	0.4061 (3)	0.86328 (16)	0.88221 (14)	0.0214 (4)	0.9178 (19)
C12	0.5056 (3)	0.96078 (18)	0.86304 (14)	0.0258 (4)	0.9178 (19)
H12	0.6311	0.9727	0.8590	0.031*	0.9178 (19)
C13	0.4191 (3)	1.04095 (18)	0.84975 (15)	0.0294 (4)	0.9178 (19)
H13	0.4862	1.1078	0.8361	0.035*	0.9178 (19)
C14	0.2354 (3)	1.02465 (17)	0.85616 (16)	0.0282 (4)	0.9178 (19)
C15	0.1377 (5)	0.92566 (19)	0.8755 (3)	0.0283 (6)	0.9178 (19)
H15	0.0120	0.9134	0.8794	0.034*	0.9178 (19)
C16	0.2227 (3)	0.8451 (2)	0.8892 (3)	0.0258 (5)	0.9178 (19)
H16	0.1563	0.7783	0.9031	0.031*	0.9178 (19)
C17	0.1398 (4)	1.11140 (18)	0.83952 (17)	0.0377 (5)	0.9178 (19)
H17A	0.1399	1.1537	0.9054	0.057*	0.9178 (19)
H17B	0.0119	1.0761	0.8008	0.057*	0.9178 (19)
H17C	0.2061	1.1604	0.8016	0.057*	0.9178 (19)
C11'	0.418 (3)	0.9037 (19)	0.8732 (19)	0.0214 (4)	0.08
C12'	0.478 (3)	1.008 (2)	0.8559 (18)	0.0258 (4)	0.08
H12'	0.6025	1.0353	0.8506	0.031*	0.0822 (19)
C13'	0.366 (3)	1.0741 (18)	0.8462 (19)	0.0294 (4)	0.08
H13'	0.4131	1.1435	0.8308	0.035*	0.0822 (19)
C14'	0.186 (3)	1.0435 (19)	0.858 (2)	0.0282 (4)	0.08
C15'	0.133 (5)	0.942 (3)	0.888 (5)	0.0283 (6)	0.08
H15'	0.0250	0.9251	0.9139	0.034*	0.0822 (19)
C16'	0.231 (4)	0.869 (2)	0.880 (4)	0.0258 (5)	0.08
H16'	0.1739	0.7942	0.8799	0.031*	0.0822 (19)
C17'	0.056 (4)	1.114 (2)	0.863 (2)	0.0377 (5)	0.08
H17D	0.1152	1.1862	0.8505	0.057*	0.0822 (19)
H17E	0.0267	1.1227	0.9297	0.057*	0.0822 (19)
H17F	−0.0591	1.0787	0.8103	0.057*	0.0822 (19)
S3	−0.00968 (5)	0.71736 (3)	0.58851 (3)	0.02206 (11)	
O1	0.43925 (15)	0.48154 (9)	0.69633 (9)	0.0210 (2)	
O2	0.52903 (18)	0.37663 (11)	0.55959 (9)	0.0296 (3)	
O3	0.35248 (16)	0.28856 (10)	0.67462 (10)	0.0288 (3)	
O7	0.16164 (16)	0.70724 (10)	0.66600 (9)	0.0237 (2)	
O8	−0.11727 (17)	0.75505 (11)	0.65271 (11)	0.0308 (3)	
O9	−0.08818 (17)	0.61688 (10)	0.51449 (10)	0.0304 (3)	
C1	0.5531 (2)	0.58641 (13)	0.68525 (13)	0.0223 (3)	
H1A	0.5740	0.5812	0.6156	0.027*	
H1B	0.6747	0.6093	0.7342	0.027*	
C2	0.4473 (2)	0.66703 (14)	0.70636 (12)	0.0230 (3)	
H2	0.5262	0.7423	0.7078	0.028*	0.9178 (19)
H2'	0.3914	0.6346	0.7594	0.028*	0.0822 (19)
C3	0.2684 (2)	0.64029 (14)	0.62715 (12)	0.0220 (3)	
H3A	0.2944	0.6568	0.5621	0.026*	
H3B	0.1993	0.5618	0.6154	0.026*	
C4	0.7047 (2)	0.39166 (13)	0.74391 (12)	0.0201 (3)	
C5	0.8713 (2)	0.44346 (14)	0.72137 (14)	0.0258 (3)	
H5	0.8721	0.4721	0.6616	0.031*	
C6	1.0356 (2)	0.45229 (15)	0.78773 (15)	0.0296 (4)	
H6	1.1499	0.4873	0.7729	0.036*	
C7	1.0371 (2)	0.41095 (14)	0.87563 (15)	0.0294 (4)	
C8	0.8685 (3)	0.36109 (16)	0.89672 (15)	0.0328 (4)	
H8	0.8677	0.3331	0.9568	0.039*	
C9	0.7014 (2)	0.35129 (15)	0.83185 (14)	0.0271 (4)	
H9	0.5871	0.3176	0.8474	0.033*	
C10	1.2193 (3)	0.41970 (18)	0.94442 (19)	0.0421 (5)	
H10A	1.2981	0.4959	0.9616	0.063*	
H10B	1.2813	0.3732	0.9096	0.063*	
H10C	1.1971	0.3958	1.0068	0.063*	
C18	0.0922 (2)	0.82229 (13)	0.53146 (12)	0.0198 (3)	
C19	0.1481 (2)	0.92984 (13)	0.58581 (13)	0.0230 (3)	
H19	0.1269	0.9462	0.6520	0.028*	
C20	0.2351 (2)	1.01301 (14)	0.54257 (14)	0.0261 (3)	
H20	0.2727	1.0867	0.5792	0.031*	
C21	0.2679 (2)	0.98960 (15)	0.44573 (14)	0.0269 (4)	
C22	0.2096 (3)	0.88154 (16)	0.39294 (14)	0.0299 (4)	
H22	0.2310	0.8649	0.3269	0.036*	
C23	0.1207 (3)	0.79717 (15)	0.43453 (13)	0.0273 (4)	
H23	0.0803	0.7236	0.3973	0.033*	
C24	0.3618 (3)	1.08080 (18)	0.39891 (17)	0.0371 (4)	
H24A	0.4166	1.1482	0.4513	0.056*	
H24B	0.2704	1.0923	0.3452	0.056*	
H24C	0.4592	1.0610	0.3698	0.056*	

### Atomic displacement parameters (Å^2^)

**Table:**

	*U*^11^	*U*^22^	*U*^33^	*U*^12^	*U*^13^	*U*^23^
S1	0.01969 (19)	0.01871 (19)	0.0219 (2)	0.00439 (15)	0.00552 (14)	0.00246 (14)
S2	0.0209 (2)	0.0224 (2)	0.0183 (2)	0.00569 (16)	0.00477 (16)	0.00529 (16)
O4	0.0244 (6)	0.0206 (6)	0.0175 (6)	0.0059 (5)	0.0077 (5)	0.0059 (5)
O5	0.0321 (7)	0.0287 (7)	0.0184 (6)	0.0096 (6)	0.0073 (5)	0.0090 (5)
O6	0.0200 (6)	0.0320 (7)	0.0309 (7)	0.0038 (5)	0.0038 (5)	0.0053 (6)
S2'	0.0209 (2)	0.0224 (2)	0.0183 (2)	0.00569 (16)	0.00477 (16)	0.00529 (16)
O4'	0.0244 (6)	0.0206 (6)	0.0175 (6)	0.0059 (5)	0.0077 (5)	0.0059 (5)
O5'	0.0321 (7)	0.0287 (7)	0.0184 (6)	0.0096 (6)	0.0073 (5)	0.0090 (5)
O6'	0.0200 (6)	0.0320 (7)	0.0309 (7)	0.0038 (5)	0.0038 (5)	0.0053 (6)
C11	0.0262 (9)	0.0190 (9)	0.0185 (8)	0.0058 (8)	0.0054 (6)	0.0032 (7)
C12	0.0289 (9)	0.0231 (10)	0.0230 (9)	0.0040 (9)	0.0067 (7)	0.0032 (7)
C13	0.0390 (12)	0.0195 (10)	0.0260 (9)	0.0042 (8)	0.0044 (8)	0.0051 (8)
C14	0.0389 (12)	0.0232 (10)	0.0186 (8)	0.0117 (8)	−0.0028 (9)	−0.0001 (7)
C15	0.0279 (9)	0.0284 (12)	0.0260 (16)	0.0091 (9)	0.0026 (8)	0.0015 (13)
C16	0.0246 (8)	0.0254 (13)	0.0268 (11)	0.0053 (9)	0.0063 (7)	0.0069 (10)
C17	0.0491 (14)	0.0290 (10)	0.0318 (11)	0.0166 (10)	−0.0024 (9)	0.0021 (8)
C11'	0.0262 (9)	0.0190 (9)	0.0185 (8)	0.0058 (8)	0.0054 (6)	0.0032 (7)
C12'	0.0289 (9)	0.0231 (10)	0.0230 (9)	0.0040 (9)	0.0067 (7)	0.0032 (7)
C13'	0.0390 (12)	0.0195 (10)	0.0260 (9)	0.0042 (8)	0.0044 (8)	0.0051 (8)
C14'	0.0389 (12)	0.0232 (10)	0.0186 (8)	0.0117 (8)	−0.0028 (9)	−0.0001 (7)
C15'	0.0279 (9)	0.0284 (12)	0.0260 (16)	0.0091 (9)	0.0026 (8)	0.0015 (13)
C16'	0.0246 (8)	0.0254 (13)	0.0268 (11)	0.0053 (9)	0.0063 (7)	0.0069 (10)
C17'	0.0491 (14)	0.0290 (10)	0.0318 (11)	0.0166 (10)	−0.0024 (9)	0.0021 (8)
S3	0.01781 (19)	0.0201 (2)	0.0277 (2)	0.00455 (14)	0.00356 (15)	0.00722 (15)
O1	0.0194 (5)	0.0187 (5)	0.0269 (6)	0.0051 (4)	0.0098 (4)	0.0065 (4)
O2	0.0333 (7)	0.0351 (7)	0.0205 (6)	0.0119 (5)	0.0077 (5)	0.0015 (5)
O3	0.0222 (6)	0.0202 (6)	0.0395 (7)	0.0006 (5)	0.0050 (5)	0.0051 (5)
O7	0.0247 (6)	0.0274 (6)	0.0212 (6)	0.0126 (5)	0.0034 (4)	0.0055 (5)
O8	0.0252 (6)	0.0324 (7)	0.0424 (7)	0.0118 (5)	0.0156 (5)	0.0157 (6)
O9	0.0252 (6)	0.0183 (6)	0.0396 (7)	0.0018 (5)	−0.0047 (5)	0.0041 (5)
C1	0.0182 (7)	0.0203 (8)	0.0282 (8)	0.0037 (6)	0.0061 (6)	0.0071 (6)
C2	0.0240 (8)	0.0219 (8)	0.0228 (8)	0.0073 (6)	0.0046 (6)	0.0043 (6)
C3	0.0233 (8)	0.0237 (8)	0.0217 (8)	0.0110 (6)	0.0047 (6)	0.0067 (6)
C4	0.0186 (7)	0.0178 (7)	0.0240 (8)	0.0056 (6)	0.0056 (6)	0.0035 (6)
C5	0.0242 (8)	0.0235 (8)	0.0318 (9)	0.0063 (7)	0.0120 (7)	0.0071 (7)
C6	0.0217 (8)	0.0249 (8)	0.0428 (10)	0.0054 (7)	0.0114 (7)	0.0069 (7)
C7	0.0239 (8)	0.0223 (8)	0.0393 (10)	0.0086 (7)	0.0012 (7)	0.0027 (7)
C8	0.0331 (9)	0.0344 (10)	0.0337 (10)	0.0113 (8)	0.0065 (8)	0.0145 (8)
C9	0.0253 (8)	0.0278 (8)	0.0297 (9)	0.0054 (7)	0.0099 (7)	0.0104 (7)
C10	0.0300 (10)	0.0362 (11)	0.0558 (13)	0.0129 (8)	−0.0044 (9)	0.0083 (9)
C18	0.0170 (7)	0.0202 (7)	0.0213 (7)	0.0056 (6)	0.0020 (6)	0.0049 (6)
C19	0.0214 (7)	0.0221 (8)	0.0228 (8)	0.0037 (6)	0.0055 (6)	0.0015 (6)
C20	0.0225 (8)	0.0199 (8)	0.0345 (9)	0.0047 (6)	0.0067 (7)	0.0045 (7)
C21	0.0230 (8)	0.0307 (9)	0.0342 (9)	0.0131 (7)	0.0096 (7)	0.0155 (7)
C22	0.0369 (9)	0.0356 (10)	0.0243 (8)	0.0171 (8)	0.0119 (7)	0.0107 (7)
C23	0.0338 (9)	0.0236 (8)	0.0240 (8)	0.0116 (7)	0.0040 (7)	0.0019 (6)
C24	0.0329 (10)	0.0400 (11)	0.0500 (12)	0.0156 (8)	0.0172 (9)	0.0267 (9)

### Geometric parameters (Å, º)

**Table:**

S1—O3	1.4241 (13)	S3—O8	1.4258 (13)
S1—O2	1.4309 (12)	S3—O9	1.4308 (13)
S1—O1	1.5792 (11)	S3—O7	1.5848 (12)
S1—C4	1.7574 (16)	S3—C18	1.7506 (16)
S2—O5	1.4276 (13)	O1—C1	1.4581 (18)
S2—O6	1.4268 (14)	O7—C3	1.4478 (19)
S2—O4	1.5853 (13)	C1—C2	1.508 (2)
S2—C11	1.751 (2)	C1—H1A	0.9900
O4—C2	1.4424 (19)	C1—H1B	0.9900
S2'—O5'	1.432 (9)	C2—C3	1.513 (2)
S2'—O6'	1.433 (8)	C2—H2	1.0000
S2'—O4'	1.573 (7)	C2—H2'	1.0000
S2'—C11'	1.68 (2)	C3—H3A	0.9900
O4'—C2	1.382 (8)	C3—H3B	0.9900
C11—C12	1.386 (3)	C4—C9	1.387 (2)
C11—C16	1.391 (3)	C4—C5	1.394 (2)
C12—C13	1.390 (3)	C5—C6	1.384 (3)
C12—H12	0.9500	C5—H5	0.9500
C13—C14	1.393 (3)	C6—C7	1.391 (3)
C13—H13	0.9500	C6—H6	0.9500
C14—C15	1.398 (3)	C7—C8	1.390 (3)
C14—C17	1.519 (3)	C7—C10	1.509 (3)
C15—C16	1.387 (3)	C8—C9	1.389 (3)
C15—H15	0.9500	C8—H8	0.9500
C16—H16	0.9500	C9—H9	0.9500
C17—H17A	0.9800	C10—H10A	0.9800
C17—H17B	0.9800	C10—H10B	0.9800
C17—H17C	0.9800	C10—H10C	0.9800
C11'—C12'	1.382 (18)	C18—C23	1.387 (2)
C11'—C16'	1.416 (18)	C18—C19	1.390 (2)
C12'—C13'	1.370 (18)	C19—C20	1.387 (2)
C12'—H12'	0.9500	C19—H19	0.9500
C13'—C14'	1.377 (17)	C20—C21	1.396 (3)
C13'—H13'	0.9500	C20—H20	0.9500
C14'—C15'	1.412 (18)	C21—C22	1.388 (3)
C14'—C17'	1.529 (17)	C21—C24	1.511 (2)
C15'—C16'	1.365 (18)	C22—C23	1.389 (3)
C15'—H15'	0.9500	C22—H22	0.9500
C16'—H16'	0.9500	C23—H23	0.9500
C17'—H17D	0.9800	C24—H24A	0.9800
C17'—H17E	0.9800	C24—H24B	0.9800
C17'—H17F	0.9800	C24—H24C	0.9800
			
O3—S1—O2	120.39 (8)	O1—C1—C2	106.13 (12)
O3—S1—O1	104.08 (7)	O1—C1—H1A	110.5
O2—S1—O1	108.72 (7)	C2—C1—H1A	110.5
O3—S1—C4	109.80 (8)	O1—C1—H1B	110.5
O2—S1—C4	108.35 (8)	C2—C1—H1B	110.5
O1—S1—C4	104.29 (7)	H1A—C1—H1B	108.7
O5—S2—O6	120.42 (8)	O4'—C2—C1	119.5 (6)
O5—S2—O4	103.08 (7)	O4—C2—C1	108.20 (13)
O6—S2—O4	108.83 (8)	O4'—C2—C3	124.5 (6)
O5—S2—C11	109.48 (8)	O4—C2—C3	108.88 (13)
O6—S2—C11	109.22 (9)	C1—C2—C3	111.91 (14)
O4—S2—C11	104.56 (8)	O4—C2—H2	109.3
C2—O4—S2	120.41 (10)	C1—C2—H2	109.3
O5'—S2'—O6'	118.3 (9)	C3—C2—H2	109.3
O5'—S2'—O4'	109.3 (7)	O7—C3—C2	106.89 (13)
O6'—S2'—O4'	104.4 (7)	O7—C3—H3A	110.3
O5'—S2'—C11'	108.9 (11)	C2—C3—H3A	110.3
O6'—S2'—C11'	110.8 (11)	O7—C3—H3B	110.3
O4'—S2'—C11'	104.1 (10)	C2—C3—H3B	110.3
C2—O4'—S2'	135.1 (8)	H3A—C3—H3B	108.6
C12—C11—C16	121.07 (18)	C9—C4—C5	121.26 (16)
C12—C11—S2	120.16 (15)	C9—C4—S1	119.20 (13)
C16—C11—S2	118.77 (15)	C5—C4—S1	119.53 (13)
C11—C12—C13	119.08 (18)	C6—C5—C4	118.72 (16)
C11—C12—H12	120.5	C6—C5—H5	120.6
C13—C12—H12	120.5	C4—C5—H5	120.6
C12—C13—C14	120.92 (18)	C5—C6—C7	121.40 (16)
C12—C13—H13	119.5	C5—C6—H6	119.3
C14—C13—H13	119.5	C7—C6—H6	119.3
C15—C14—C13	119.0 (2)	C8—C7—C6	118.50 (16)
C15—C14—C17	120.1 (2)	C8—C7—C10	121.84 (18)
C13—C14—C17	120.9 (2)	C6—C7—C10	119.65 (18)
C16—C15—C14	120.6 (2)	C7—C8—C9	121.46 (17)
C16—C15—H15	119.7	C7—C8—H8	119.3
C14—C15—H15	119.7	C9—C8—H8	119.3
C15—C16—C11	119.3 (2)	C4—C9—C8	118.64 (16)
C15—C16—H16	120.3	C4—C9—H9	120.7
C11—C16—H16	120.3	C8—C9—H9	120.7
C12'—C11'—C16'	115.4 (17)	C7—C10—H10A	109.5
C12'—C11'—S2'	120.4 (18)	C7—C10—H10B	109.5
C16'—C11'—S2'	124.2 (18)	H10A—C10—H10B	109.5
C11'—C12'—C13'	122.9 (18)	C7—C10—H10C	109.5
C11'—C12'—H12'	118.6	H10A—C10—H10C	109.5
C13'—C12'—H12'	118.6	H10B—C10—H10C	109.5
C12'—C13'—C14'	122.4 (18)	C23—C18—C19	120.99 (15)
C12'—C13'—H13'	118.8	C23—C18—S3	119.93 (13)
C14'—C13'—H13'	118.8	C19—C18—S3	119.05 (12)
C13'—C14'—C15'	114.4 (18)	C20—C19—C18	119.37 (15)
C13'—C14'—C17'	128 (2)	C20—C19—H19	120.3
C15'—C14'—C17'	116.9 (19)	C18—C19—H19	120.3
C16'—C15'—C14'	122 (2)	C19—C20—C21	120.68 (16)
C14'—C15'—H15'	118.8	C19—C20—H20	119.7
C15'—C16'—C11'	120 (2)	C21—C20—H20	119.7
C15'—C16'—H16'	120.1	C22—C21—C20	118.71 (16)
C11'—C16'—H16'	120.1	C22—C21—C24	120.93 (17)
C14'—C17'—H17D	109.5	C20—C21—C24	120.34 (17)
C14'—C17'—H17E	109.5	C21—C22—C23	121.48 (16)
H17D—C17'—H17E	109.5	C21—C22—H22	119.3
C14'—C17'—H17F	109.5	C23—C22—H22	119.3
H17D—C17'—H17F	109.5	C18—C23—C22	118.74 (16)
H17E—C17'—H17F	109.5	C18—C23—H23	120.6
O8—S3—O9	120.40 (8)	C22—C23—H23	120.6
O8—S3—O7	103.60 (7)	C21—C24—H24A	109.5
O9—S3—O7	108.61 (7)	C21—C24—H24B	109.5
O8—S3—C18	109.66 (8)	H24A—C24—H24B	109.5
O9—S3—C18	110.02 (8)	C21—C24—H24C	109.5
O7—S3—C18	102.96 (7)	H24A—C24—H24C	109.5
C1—O1—S1	118.12 (9)	H24B—C24—H24C	109.5
C3—O7—S3	117.53 (10)		
			
O5—S2—O4—C2	−167.25 (12)	S2'—O4'—C2—O4	0.7 (11)
O6—S2—O4—C2	−38.32 (14)	S2'—O4'—C2—C1	−101.6 (12)
C11—S2—O4—C2	78.29 (13)	S2'—O4'—C2—C3	103.1 (12)
O5'—S2'—O4'—C2	13.9 (16)	S2—O4—C2—O4'	−9.5 (6)
O6'—S2'—O4'—C2	141.3 (13)	S2—O4—C2—C1	107.04 (13)
C11'—S2'—O4'—C2	−102.3 (15)	S2—O4—C2—C3	−131.12 (12)
O5—S2—C11—C12	136.50 (15)	O1—C1—C2—O4'	136.3 (5)
O6—S2—C11—C12	2.71 (18)	O1—C1—C2—O4	54.53 (16)
O4—S2—C11—C12	−113.63 (15)	O1—C1—C2—C3	−65.43 (17)
O5—S2—C11—C16	−43.0 (2)	S3—O7—C3—C2	165.70 (10)
O6—S2—C11—C16	−176.8 (2)	O4'—C2—C3—O7	−34.9 (5)
O4—S2—C11—C16	66.9 (2)	O4—C2—C3—O7	48.53 (16)
C16—C11—C12—C13	−0.6 (3)	C1—C2—C3—O7	168.09 (13)
S2—C11—C12—C13	179.93 (15)	O3—S1—C4—C9	−21.13 (16)
C11—C12—C13—C14	0.4 (3)	O2—S1—C4—C9	−154.44 (14)
C12—C13—C14—C15	−0.5 (3)	O1—S1—C4—C9	89.88 (14)
C12—C13—C14—C17	−178.76 (19)	O3—S1—C4—C5	158.15 (13)
C13—C14—C15—C16	0.6 (5)	O2—S1—C4—C5	24.85 (15)
C17—C14—C15—C16	178.9 (3)	O1—S1—C4—C5	−90.84 (14)
C14—C15—C16—C11	−0.7 (5)	C9—C4—C5—C6	1.3 (3)
C12—C11—C16—C15	0.7 (4)	S1—C4—C5—C6	−177.99 (13)
S2—C11—C16—C15	−179.8 (3)	C4—C5—C6—C7	−0.1 (3)
O5'—S2'—C11'—C12'	158 (2)	C5—C6—C7—C8	−0.8 (3)
O6'—S2'—C11'—C12'	26 (2)	C5—C6—C7—C10	178.39 (17)
O4'—S2'—C11'—C12'	−85 (2)	C6—C7—C8—C9	0.5 (3)
O5'—S2'—C11'—C16'	−24 (3)	C10—C7—C8—C9	−178.60 (18)
O6'—S2'—C11'—C16'	−156 (3)	C5—C4—C9—C8	−1.5 (3)
O4'—S2'—C11'—C16'	92 (3)	S1—C4—C9—C8	177.78 (14)
C16'—C11'—C12'—C13'	0 (4)	C7—C8—C9—C4	0.6 (3)
S2'—C11'—C12'—C13'	178 (2)	O8—S3—C18—C23	−147.39 (14)
C11'—C12'—C13'—C14'	3 (4)	O9—S3—C18—C23	−12.79 (16)
C12'—C13'—C14'—C15'	4 (5)	O7—S3—C18—C23	102.83 (14)
C12'—C13'—C14'—C17'	172 (3)	O8—S3—C18—C19	34.45 (15)
C13'—C14'—C15'—C16'	−16 (7)	O9—S3—C18—C19	169.05 (12)
C17'—C14'—C15'—C16'	175 (5)	O7—S3—C18—C19	−75.33 (14)
C14'—C15'—C16'—C11'	20 (8)	C23—C18—C19—C20	−0.5 (2)
C12'—C11'—C16'—C15'	−11 (6)	S3—C18—C19—C20	177.62 (12)
S2'—C11'—C16'—C15'	171 (4)	C18—C19—C20—C21	−0.5 (3)
O3—S1—O1—C1	−176.79 (11)	C19—C20—C21—C22	0.8 (3)
O2—S1—O1—C1	−47.32 (13)	C19—C20—C21—C24	179.63 (16)
C4—S1—O1—C1	68.10 (12)	C20—C21—C22—C23	−0.2 (3)
O8—S3—O7—C3	161.56 (11)	C24—C21—C22—C23	−179.03 (17)
O9—S3—O7—C3	32.45 (13)	C19—C18—C23—C22	1.1 (3)
C18—S3—O7—C3	−84.18 (12)	S3—C18—C23—C22	−177.02 (13)
S1—O1—C1—C2	168.60 (10)	C21—C22—C23—C18	−0.7 (3)

### Hydrogen-bond geometry (Å, º)

**Table:**

*D*—H···*A*	*D*—H	H···*A*	*D*···*A*	*D*—H···*A*
C1—H1*A*···O2^i^	0.99	2.58	3.430 (2)	144
C3—H3*A*···O2^i^	0.99	2.39	3.230 (2)	143
C3—H3*B*···O9^ii^	0.99	2.53	3.377 (2)	144
C6—H6···O4^iii^	0.95	2.51	3.332 (2)	145
C9—H9···O5^iv^	0.95	2.50	3.166 (2)	127
C15—H15···O6^v^	0.95	2.53	3.409 (4)	154
C20—H20···O3^vi^	0.95	2.60	3.545 (2)	176
C24—H24*B*···O8^vii^	0.98	2.55	3.263 (3)	129

Symmetry codes: (i) −*x*+1, −*y*+1, −*z*+1; (ii) −*x*, −*y*+1, −*z*+1; (iii) *x*+1, *y*, *z*; (iv) −*x*+1, −*y*+1, −*z*+2; (v) *x*−1, *y*, *z*; (vi) *x*, *y*+1, *z*; (vii) −*x*, −*y*+2, −*z*+1.
